# The Sdp-SH3b2 domain contained in *Lactobacillus johnsonii* N6.2-derived extracellular vesicles inhibit murine norovirus replication

**DOI:** 10.3389/fimmu.2024.1490755

**Published:** 2024-12-05

**Authors:** Danilo R. da Silva, Asra B. Sharjeel, Reagan Beliakoff, Leandro D. Teixeira, Peter E. Kima, Melissa K. Jones, Claudio F. Gonzalez, Graciela L. Lorca

**Affiliations:** ^1^ Department of Microbiology and Cell Science, Genetics Institute, Institute of Food and Agricultural Sciences, University of Florida, Gainesville, FL, United States; ^2^ Department of Microbiology and Cell Science, Institute of Food and Agricultural Sciences, University of Florida, Gainesville, FL, United States

**Keywords:** Lactobacillus johnsonii N6.2, probiotic, MNV-1, bacterial effector, extracellular vesicle, SH3b

## Abstract

The internalization of *Lactobacillus johnsonii* N6.2 extracellular vesicles (EVs) by cells results in a significant induction of the 2’,5’-oligoadenylate synthetase (OAS) pathway. It also induces expression of *IFI44L, MX1, MX2* and *DDX60*. In this work, we evaluated whether the antiviral response induced by *L. johnsonii* N6.2-derived EVs, has an inhibitory effect on an RNA viral insult using murine norovirus (MNV-1) as the viral infection model. We found that RAW 264.7 Macrophages treated with EVs significantly decreased the levels of MNV-1 genome. These results were consistent with an increase in expression of *Oas1b, Oas2, Oasl, Mx1, Mx2* and *Ifi44l* (6 hours post infection). Out of six proteins enriched in EVs, we found that SH3b2 domain of Sdp was the only protein effector molecule able to recapitulate the activation of the OAS pathway. In C57BL6 mice, the administration of live *L. johnsonii* N6.2, EVs, and Sdp-SH3b2/liposomes significantly decreased MNV-1 titers in the distal ileum, in contrast to the controls with PBS and liposomes alone that did not affect MNV-1. These results establish that the SH3b2 domain of Sdp, which is enriched in *L. johnsonii* derived EVs, is an effector molecule in EVs that can orchestrate the control of viral infections *in vivo*.

## Introduction

The importance of the microbiota as modulators of host immune responses has been established (1–3). However, a significant gap in knowledge is the identity of bacterial effector molecules that mediate specific immunological effects in the host. Bacterial extracellular vesicles (EVs) have increasingly been in the forefront of research as mediators of host:microbe interactions; EVs are ubiquitously produced in all domains of life (4–8). EVs derived from commensal microbiota account for the largest and most constant interactions within the host. Therefore, there is a significant need to elucidate the mechanisms involved in commensal and probiotic bacteria derived EVs’ participation in interkingdom communication with the host.

We recently showed that *Lactobacillus johnsonii* N6.2 releases EVs with a distinct composition of proteins, and lipids when compared to whole cells (9). *L. johnsonii* N6.2 is a probiotic bacterium that has been shown to mitigate type 1 diabetes in prone rodents (BBDP rat model) by maintaining euglycemic levels and reducing the inflammatory state (10). We hypothesized that EVs play a central role in delivering bioactive molecules that may act as mechanistic effectors in immune modulation. We observed that the addition of EVs to the human pancreatic cell line βlox5 reduced cytokine-induced apoptosis (11). The role of EVs on beta cell function was further evaluated using primary human pancreatic islets. It was found that EVs significantly induced insulin secretion in the presence of high glucose concentrations. Through RNAseq analyses, a significant induction of the AHR and 2’, 5’-oligoadenylate synthetase (OAS) pathways were observed (11). The OAS pathway is part of an innate immune response activated by viral or bacterial RNA (12–14). In mammals, the OAS family is composed of three enzymatically active enzymes, OAS1, OAS2 and OAS3, all of which were significantly induced in the presence of EVs, but not by purified membranes from *L. johnsonii* N6.2 (11). Similar results were observed on the enzymatically inactive OASL, which is induced by RIG-1 and MAVS oligomerization and is crucial in stabilizing the OAS complex. Using partially purified EV components, we found that RNA present in EVs are sensed by the βlox5 cells albeit surface proteins in the EV are required for uptake into eukaryotic cells (13). Other genes involved in the sensing and response to nucleic acids, including *IFI44L, MX1, MX2* and *DDX60*, were also induced in the presence of EVs (11, 13).

The OAS pathway plays a crucial role in the cells’ antiviral response by creating 2′, 5′-oligoadenylates that can activate the latent cellular RNase L causing the degradation of the RNA viral genome (12, 15). To prevent the host response, viruses like Influenza A viruses and reoviruses have developed strategies to overcome the OAS pathway antiviral effects. For example, the NS1 protein of Influenza and the σ3 outer capsid protein of reoviruses bind double stranded RNA inhibiting OAS activation (16, 17). Similarly, it has been shown that the VF1 protein in murine norovirus (MNV-1) can inhibit the activation of the innate immune response by delaying the expression of the key IFN-β; however, the underlying mechanisms of this inhibition have not been elucidated (18).

The antiviral effects of some strains of probiotics have been reported. The mechanisms described include general responses such as pathogen exclusion, decrease in immune barrier permeability and the induction in the expression of innate immunity genes (19). For example, the administration of whole cells of *L. gasseri* SBT2055 induced the expression of *Oas1a* and *Mx1* in a murine model aiding in the clearance of the Influenza virus (20). Other probiotics such as, *Lacticaseibacillus rhamnosus* M21, *L. acidophilus, Limosilactobacillus reuteri, Ligilactobacillus salivarius* and *Bifidobacterium bifidum*, controlled Influenza viral infections by inducing the expression of cytokines such as IL1β, IL6, IFNα and IFNγ (21–23). Those studies that were performed with the whole bacterium did not identify the mechanism by which these probiotics exerted their antiviral effects. Moreover, scarce information is available on the bacterial components that mediate these antiviral affects. On this regard, *L. crispatus* BC3 and *L. gasseri* BC13 derived EVs have been proposed to decrease HIV virus attachment to target cell, which diminishes viral infection (24). Similarly, EVs of the commensals *Enterobacter cloacae* and *Bacteroides thetaiotaomicron* were shown to reduce the replication of murine norovirus through the induction of antiviral cytokines IL6, TNFα, IL1β and IFNγ (25). A recent study showed that the DNA from commensal *Escherichia coli* strain EVs can activate the cGAS-STING pathway to induce an antiviral response in mice (26).

We hypothesized that the activation of the OAS pathway by specific effector molecules present in *L. johnsonii* N6.2 secreted EVs could hinder a viral infection *in vivo*. To test this hypothesis, MNV-1 was used as an RNA virus model of infection. Considering that *L. johnsonii* N6.2 is a microorganism that resides and shed its EVs in the gastrointestinal tract, we propose that infection by MNV-1 is a biologically relevant model of infection. Based on our previous proteomic analyses, we propose that a molecule that is enriched in EVs secreted *L. johnsonii* EVs, acts as the mediator of the antiviral effects observed. Our findings are significant since norovirus accounts for 21 million cases of gastrointestinal illness in the United States annually (27). The identification of effector proteins secreted by members of the microbiota will allow the development of therapies to increase the innate immune responses in at risk individuals.

## Results

### *L. johnsonii* N6.2 EV-mediated antiviral response reduced the replication of MNV-1 and cytotoxicity in RAW 264.7 macrophages

**Figure 1:**
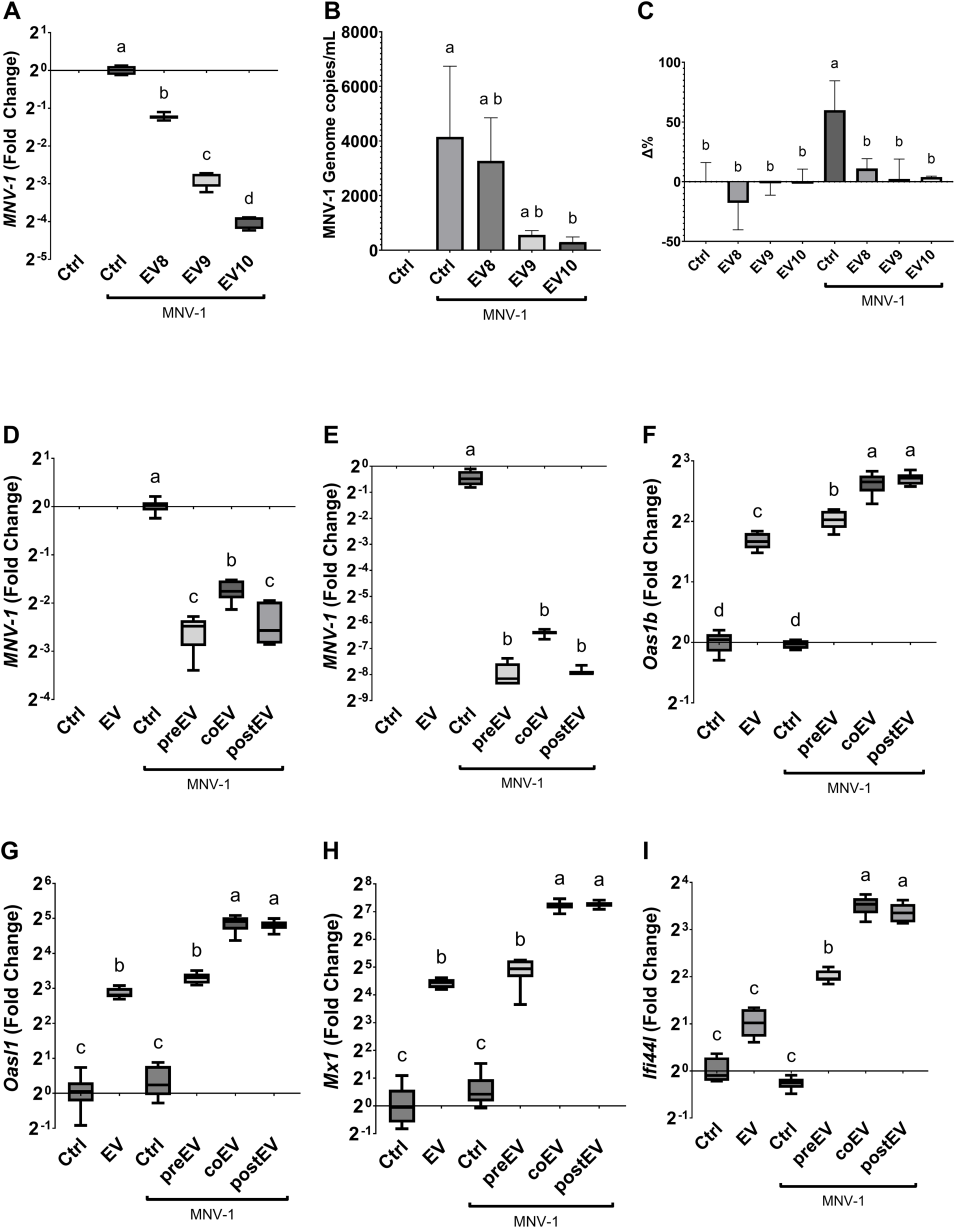
*L. johnsonii* N6.2 secreted EVs inhibit MNV replication in RAW 264.7. **(A, B)** Relative copies of MNV genome was determined by qRT-PCR at 18 hpi either in host cells **(A)** or RNA released into the supernatant **(B)**. **(C)** LHD quantification in supernatant. Activity is expressed as the percentage change (Δ%) with respect to the untreated control. Next, the effect of adding the EVs before (pre), with (co), or after (post) MNV inoculation was evaluated on MNV replication either after **(D)** 6 hpi and **(E)** 18 hpi in RAW 264.7 cells. **(F-I)** The analysis of expression levels of *Oas1b*, *Oasl1*, *Mx1* and *Ifi44l* was performed by qRT-PCR 6 hpi. Different letters on top of each bar indicates statistical significance of p ≤ 0.05 from ANOVA analysis and *post-hoc* Tukey test performed on at least three biological replicates (with two qRT-PCR technical replicates each).

First, we evaluated whether *L. johnsonii* N6.2 EVs can elicit an antiviral response in the murine RAW 264.7 Macrophages (Mφ) cell line as previously observed in human THP1 cells (11). To this end RAW 264.7 cells were treated with increasing concentrations of EVs (1 x 10^8^ EVs/mL, EV8; 1 x 10^9^ EVs/mL, EV9; 1 x 10^10^ EVs/mL, EV10) and incubated for 6 hours (h). We found that the expression of *Oas1b* (the murine *OAS1* homolog), *Oas2*, *Oasl, Mx1* were significantly increased in presence of EVs, in a dose dependent manner. Moreover, the RAW 264.7 cells also upregulated the expression of *Ifi44l*, in agreement with our previous results in human cell lines in response to EVs (**Supplementary Figure S1**). Next, we evaluated whether the observed antiviral response induced by *L. johnsonii* N6.2-derived EVs has an inhibitory effect against an RNA virus infection, using the MNV-1 as the viral infection model. MNV-1 is a positive-sense RNA virus that can infect murine Mφ cell lines like RAW 264.7 (25). To evaluate the effect of EVs on MNV-1 infection, RAW 264.7 cells were challenged with MNV-1 in the presence or absence of increasing concentrations of EVs. The relative replication level of the MNV-1 genome was assessed by qRT-PCR at 18 hours post infection (hpi) in Mφ cells, and the viral titer shed was quantified from the supernatant (**Figure 1A, B**). We found that RAW 264.7 Mφs that were pretreated with EVs significantly decreased the amount of MNV-1 genome replication in a dose dependent manner, both in Mφ cells as well as in the supernatant. The highest inhibitory effect was observed using EV10.

It was expected that a decrease in viral genome replication mediated by EVs would be correlated with a decrease in viral induced cytotoxicity and lysis. To this end, lactate dehydrogenase (LDH) activity was measured. RAW 264.7 cells challenged with MNV-1 in presence or absence of increasing concentrations of EVs and LDH activity evaluated. Compared to the no infection control, there was approximately a 60.0% (± 24.5%, p=0.0042) increase in LDH activity in the supernatant of MNV infection control (**Figure 1C**). Interestingly, all concentrations of EVs were able to prevent cell lysis. These findings, combined with the decrease in both cellular and supernatant viral load, indicated that the antiviral response induced by the *L. johnsonii* N6.2 EVs prevented viral induced cytotoxicity by decreasing the overall viral genome available for virus formation and cell lysis, suppressing viral replication overall and not viral egress.

The impact of MNV-1 replication and EVs on the expression of RNA sensing pathway in Mφs was assessed after 18 hpi. The reduction in MNV replication was positively correlated with an increase in expression of *Oas1b, Oas2, Oasl, Mx1, Mx2* and *Ifi44l* at 18 hpi (**Supplementary Figure S1**). Next, we tested whether the timing of the addition of EVs had a significant effect on MNV-1 infection. EVs were either added 5 h prior to virus inoculation, co-inoculated with MNV-1, or the EVs were added 1 h after the virus inoculation. MNV-1 replication and RNA sensing pathway in the host were assessed after 6 or 18 hpi. It was found that all *L. johnsonii* N6.2 EVs treatments were equally effective at decreasing MNV-1 replication (**Figure 1D, E**). These results suggested that the time of EVs exposure does not play a crucial role in the hindrance of viral replication and that EVs may not inhibit MNV-1 entry. These results may be explained by the quick uptake of *L. johnsonii* N6.2 EVs into eukaryotic cells as recently reported (13).

To determine if a shorter incubation time would differentially affect the induction of RNA sensing genes that limit MNV replication, RAW 264.7 cells were challenged with MNV in presence or absence of increasing concentrations of EVs and they were evaluated after 6 hpi. We found that the expressions of *Oas1b*, *Oasl*, *If44l*, and *Mx1* were not induced by MNV-1 alone at 6 hpi (**Figure 1F-I**), in contrast to the results obtained after 18 hpi (**Supplementary Figure S1**). EVs alone or EVs pre-incubation for 5 h followed by MNV-1 infection showed similar levels of induction in all the host genes tested, while the addition of EVs during MNV infection or 1 h after infection resulted in significantly higher induction levels of the RNA sensing genes tested. These results indicate that the quick uptake of EVs may be responsible for eliciting the activation of the host antiviral response resulting in reduced MNV-1 replication.

### *L. johnsonii* N6.2 EVs elicit an antiviral response that triggers a tolerogenic cytokine profile

**Figure 2:**
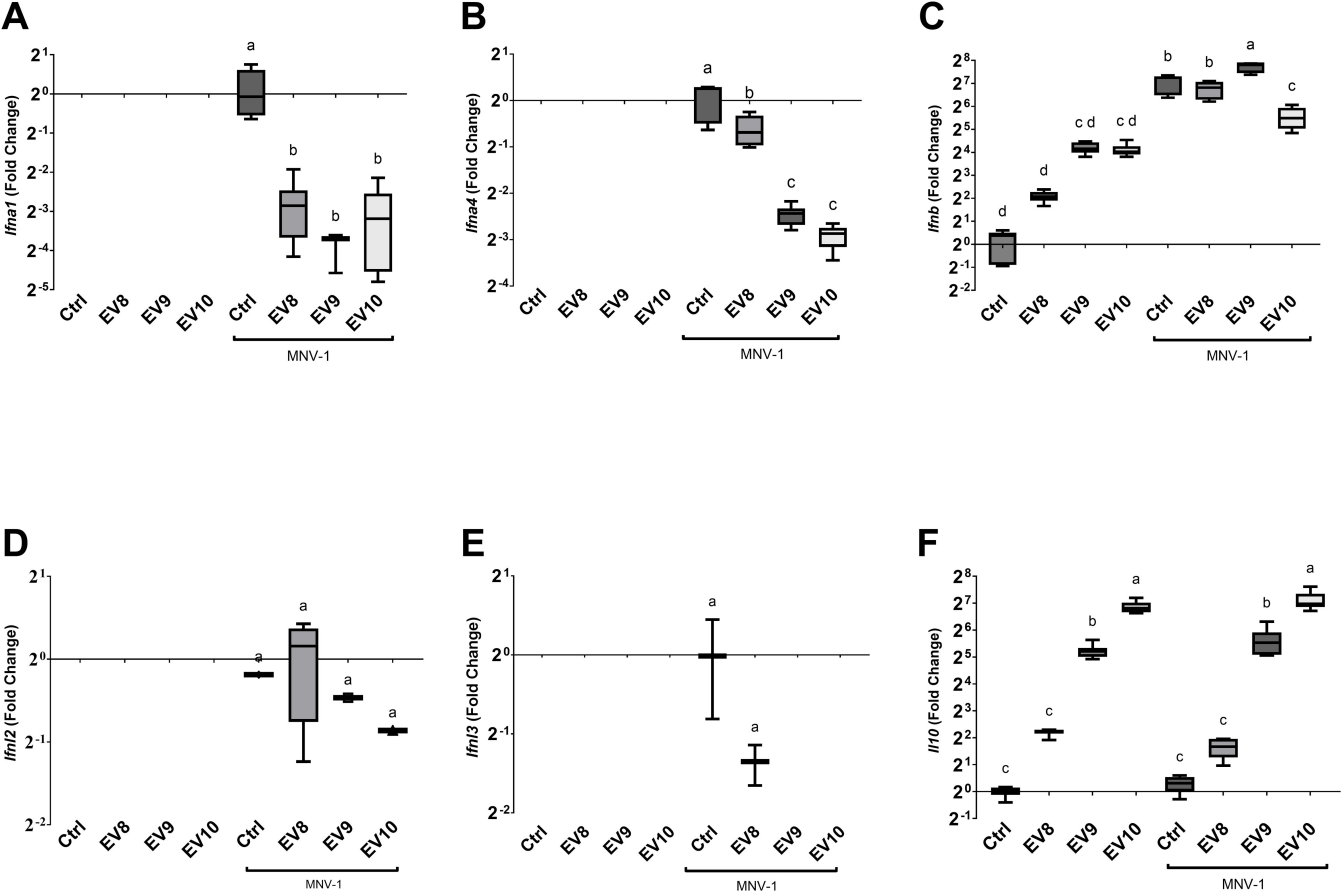
Administration of *L. johnsonii* N6.2 EVs alters the cytokine expression profile induced by MNV 18 hpi in RAW 264.7 cells. **(A-F)** Analysis of mRNA levels of the murine cytokine genes *Ifna1, Ifna4, Ifnb, Ifnl2, Ifnl3 and Il10*, respectively, by qRT-PCR. Different letters on top of each bar indicates statistical significance of p ≤ 0.05 from ANOVA analysis and *post-hoc* Tukey test performed on three biological replicates (with two qRT-PCR technical replicates each).

A canonical antiviral response culminates in the expression of type I interferons (IFN-I, IFNα and IFNβ), followed by the induction of the expression of innate immune response genes in nearby cells (28). To further elucidate the mechanisms by which the *L. johnsonii* N6.2 EVs elicits the antiviral response, the expression of murine type one interferons, IFNα (*Ifna1* and *Ifna4*) and IFNβ (*Ifnb)* genes, and type three interferons, IFNλ (*Ifnl2* and *Ifnl3*) genes, were analyzed in the presence and absence of the EVs and/or MNV-1 at 18 hpi. It was found that MNV-1 induced the expression of IFNα genes, *Ifna1* and *Ifna4, as well as Ifnb* (**Figures 2A–C**) while the other genes tested were not strongly induced by the MNV-1 infection (*Ifnl2* and *Ifnl3*) (**Figures 2D, E**). The addition of EVs alone did not result in expression of *Ifna1* and *Ifna4* while the addition of EVs during MNV-1 infection resulted in significant decrease in the expression of both genes. Noteworthy, the expression of *Ifna1* was reduced at all concentrations of EVs tested while *Ifna4* was dose dependent and linked to MNV-1 titers. In contrast, RAW 264.7 cells had basal level of expression *IFNB*. It was observed that although *Ifnb* expression increased in the presence of the EVs, but it was significantly lower than MNV-1 alone. Nonetheless, EV10 treatment of the RAW 264.7 cells inoculated with MNV-1 showed a decrease in *Ifnb* expression, as observed in the IFNα genes, when compared to the MNV-1 infection control (**Figure 2**).

The expression of IFN-I as an outcome of virus infection, can limit viral infections, but when unchecked can lead to deleterious inflammation (28, 29). We have previously reported that *L. johnsonii* N6.2-derived EVs induce IL10 secretion in human THP1 and beta cells (11, 13). Here, we found that RAW 264.7 cells have a strong dose-dependent induction of the *Il10* genes in the presence of the *L. johnsonii* N6.2 EVs while MNV-1 positive control did not induce the expression of *Il10* (**Figure 2F**). We propose that the strong induction of IL10 expression by EVs may limit the detrimental inflammatory effects produced by the induction of IFNβ expression in presence of MNV-1.

### The SH3b2 domain of Sdp stimulates innate immune responses similar to EVs

**Figure 3:**
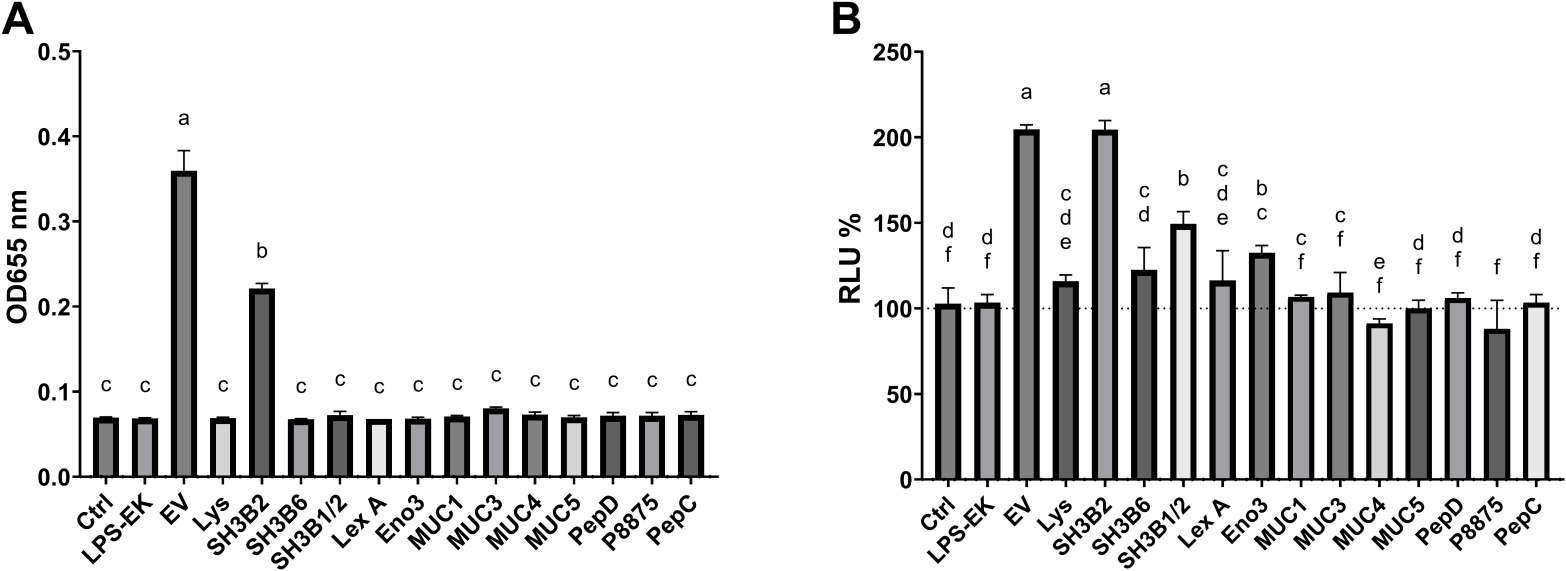
The SH3b2 domain of Sdp stimulate the expression of MIP-2 and ISRE54 promoters in the RAW-DUAL KO-TLR4 cells. Proteins enriched in EVs were purified and tested at 1.5 µg/mL in RAW-DUAL KO-TLR4 cells. After 6 h of incubation, the stimulation of the MIP-2 promoter fusion to supernatant alkaline phosphatase (SEAP) and interferon signaling (ISRE promoter fusion to Lucia luciferase) was evaluated. **(A)** SEAP activity is expressed as OD at 655 nm after 16 (h). **(B)** Relative luminescence expressed as percentage relative to LPS stimulated cells used as negative control. Different letters on top of each bar indicates statistical significance of p ≤ 0.05 from ANOVA analysis and *post-hoc* Tukey test performed on three biological replicates.

Following on our initial proteomic analyses of *L. johnsonii* EVs, we selected and purified differentially enriched proteins in EVs (9). The proteins Eno3, P1875, P8875, P1390 were purified using Ni^+^ affinity columns (**Supplementary Figure S2**). We also selected Sdp and Muc. The later proteins are composed of well characterized structure-based domains: Sdp (Lysin, SH3b1-SH3b2, SH3b2 and SH3b6) and Muc (Muc1, Muc3, Muc4 and Muc5). We proceeded to purify each of these protein domains and to test their capacity to elicit cell responses. To evaluate the impact of each of the proteins or domains on stimulation of innate immune responses, RAW-Dual™ KO-TLR4 Mφ cells were utilized. The availability of a TLR4 knockout allowed for the exclusion of effects by potential lipopolysaccharide contamination from protein purification. Additionally, the two reporter genes in Mφ allowed the quantification of the signaling through NF-κB (MIP-2 promoter fusion to supernatant alkaline phosphatase, SEAP) and interferon signaling (ISRE promoter fusion to Lucia luciferase). Each purified protein was tested at 1.5 µg/mL. Mock was prepared following a protein purification protocol using *E. coli* BL21 (DE3) not containing an expression vector. We found that of the Sdp domains, SH3b2 significantly induced the expression of the MIP-2 and ISRE to a similar level as EVs. While the Sdp-SH3b1-SH3b2 fusion and Eno had a small but significant induction in activity, the Sdp domains SH3b6 and Sdp-Lys, as well as the other proteins tested, did not induce significant changes in the Lucia luciferase activity reporter genes (**Figure 3B**). Likewise, the LPS and mock controls did not stimulate either reporter gene. EVs and Sdp-SH3b2 were the only components able to induce the expression of the SEAP reporters while all other components tested did not induce SEAP expression and activity (**Figure 3A**).

### *L. johnsonii* N6.2 EVs and SH3b2 utilize the TRIF/TRAM and MyD88 adaptor proteins pathways to initiate cellular response

**Figure 4:**
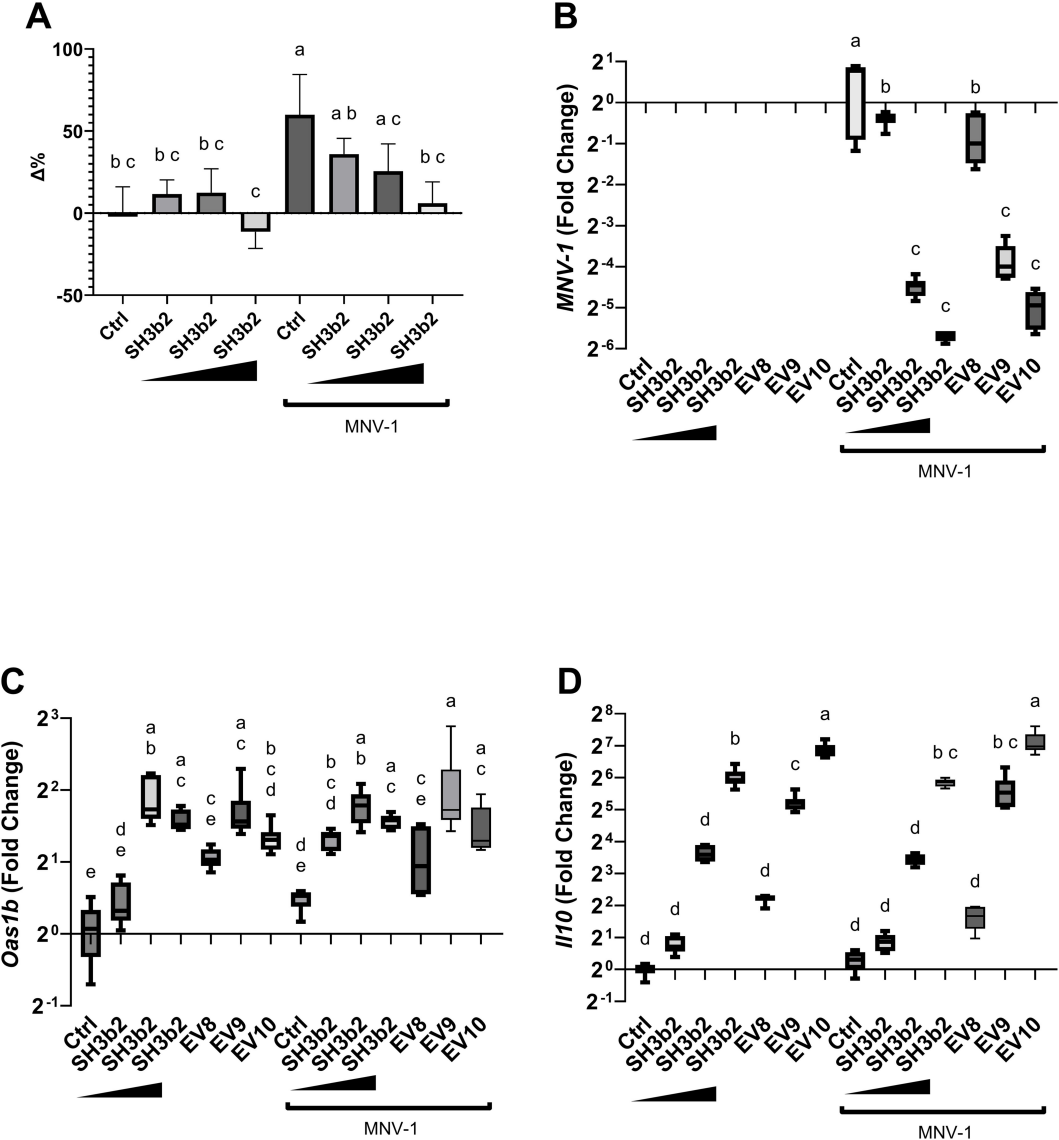
SH3b2 mitigates MNV-1 infection in a dose dependent manner. Increasing concentrations of purified SH3b2 (0.015, 0.15, and 1.5 µg/mL) were added to RAW 264.7 cells and infected with MNV-1. 6 hpi the cellular response as well as the viral levels were quantified by qRT-PCR. **(A)** Cell lysis assessed by LHD quantification in supernatant. Activity is expressed as the percentage change (Δ %) with respect to the untreated control. **(B)** Relative copies of MNV genome; as well as **(C)** *Oas1b*, and **(D)** *Il10* expression were quantified by qRT-PCR. Different letters on top of each bar indicates statistical significance of p ≤ 0.05 from ANOVA analysis and *post-hoc* Tukey test performed on three biological replicates (with two qRT-PCR technical replicates each).

**Figure 5:**
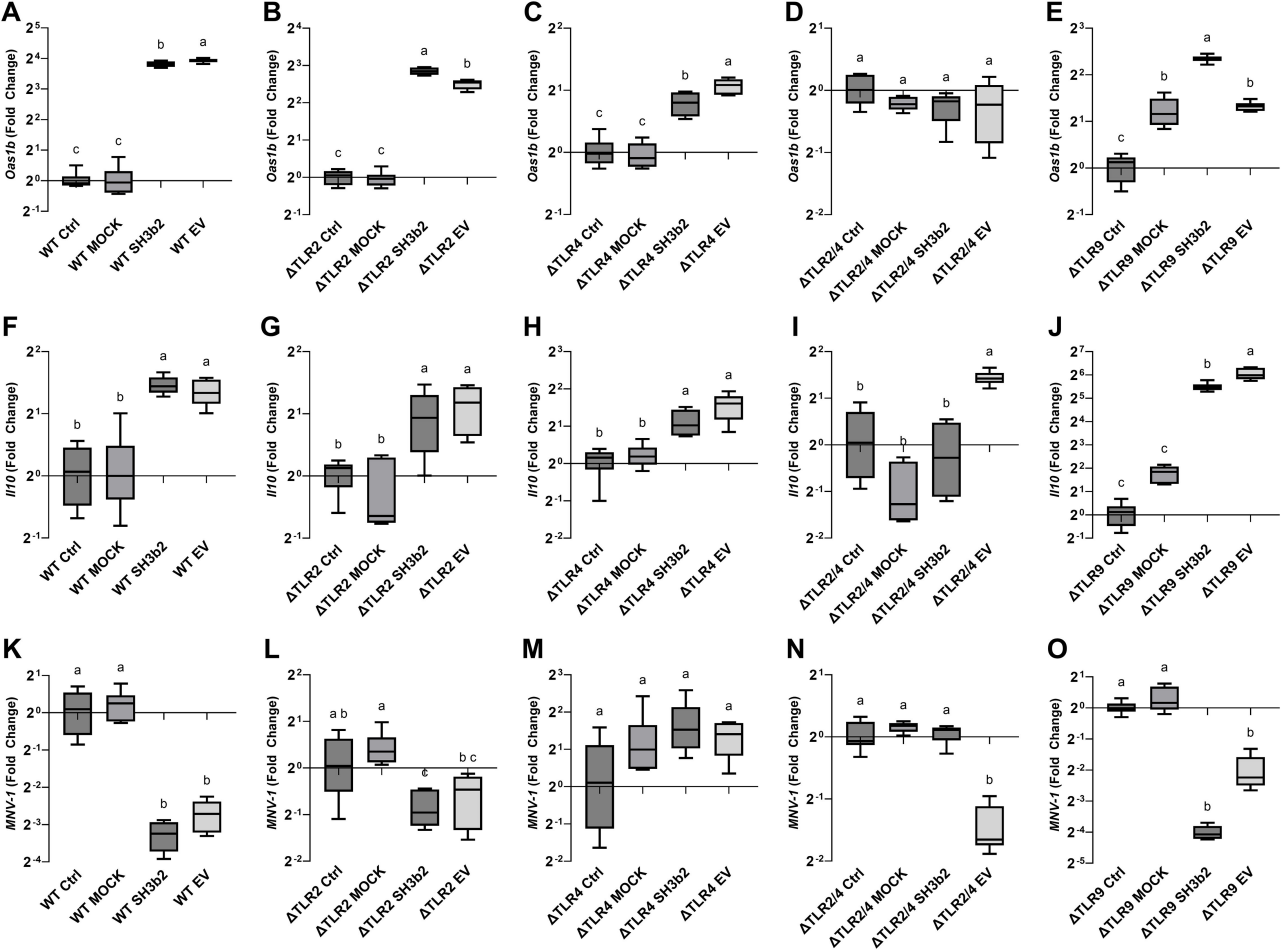
SH3b2 and EVs mitigates MNV-1 infection by stimulation of pathways mediated by the ΔTLR2/TLR4. Purified SH3b2 (1.5 µg/mL), EV10, and purification Mock (MOCK) and buffer controls were added to murine Mφ cells NR-9456. The stimulation of the expression of the mRNA levels of *Oas1b* and *Il10* genes was evaluated after 6 h in wild type murine Mφs **(A, F)** as well as in Mφs derived from knockout mice in the ΔTLR2 **(B, G)**, ΔTLR4 **(C, H)**, ΔTLR2/TLR4 **(D, I)**, and ΔTLR9 **(E, J)**, respectively. The same set up was infected with MNV-1 (**K-O** respectively), and the MNV-1 genome titer was quantified 18 hpi by qRT-PCR. Different letters on top of each bar indicates statistical significance of p ≤ 0.05 from ANOVA analysis and *post-hoc* Tukey test performed on three biological replicates (with two qRT-PCR technical replicates each).

**Figure 6:**
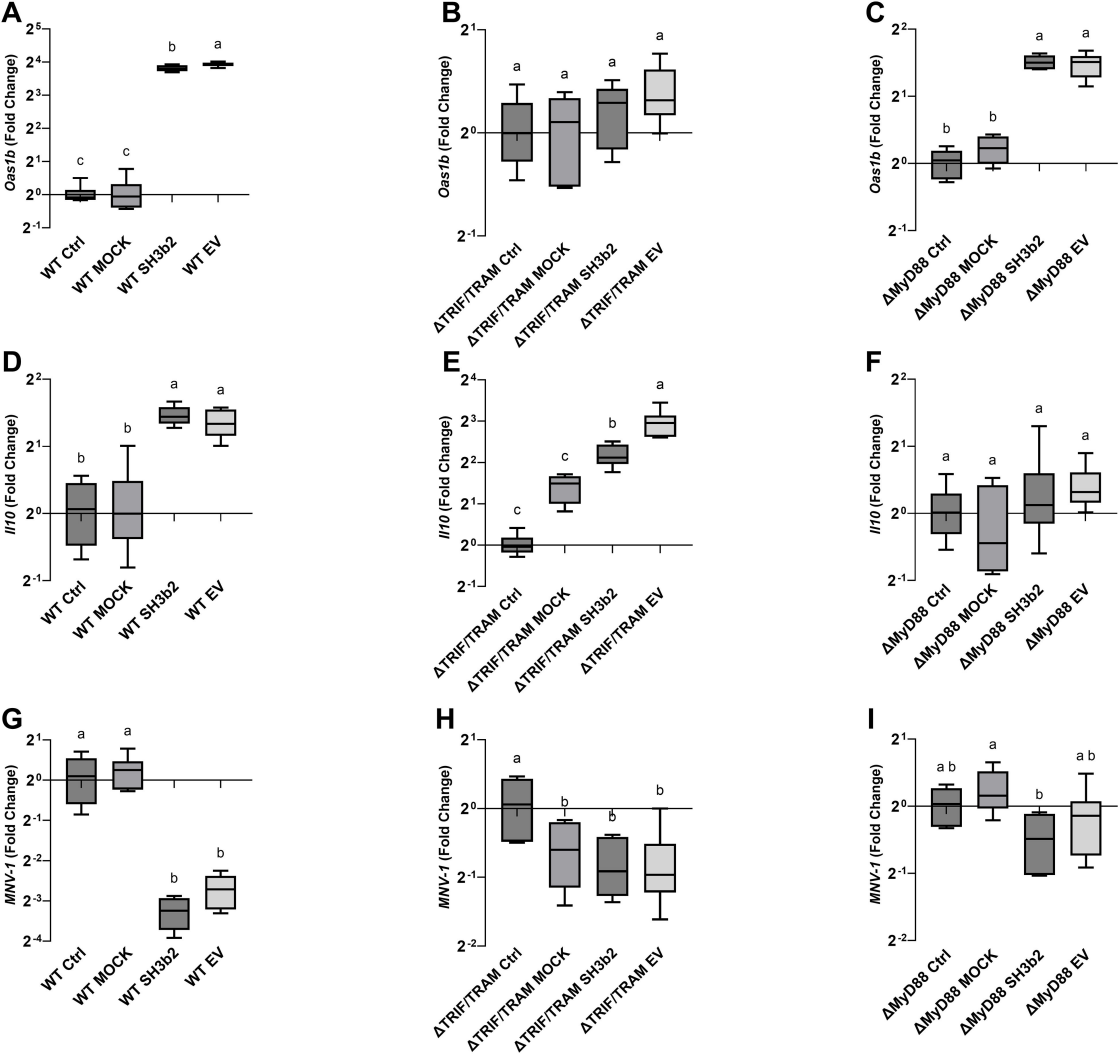
SH3b2 and EVs mitigates MNV-1 infection by stimulation of pathways mediated by the ΔTRIF/TRAM and ΔMyD88 adaptors. Purified SH3b2 (1.5 µg/mL), EV10, and purification MOCK and buffer controls were added to immortalized murine Mφ cells. The stimulation of the expression of the mRNA levels of *Oas1b* and *Il10* genes was evaluated after 6 h in wild type murine Mφs **(A, D)** as well as in Mφs derived from knockout mice in the ΔTRIF/TRAM **(B, E)**, and ΔMyD88 **(C, F)**, respectively. The same set up was infected with MNV-1 (**G–I**, respectively), and the MNV-1 genome titer was quantified 18 hpi by qRT-PCR. Different letters on top of each bar indicates statistical significance of p ≤ 0.05 from ANOVA analysis and *post-hoc* Tukey test performed on three biological replicates (with two qRT-PCR technical replicates each).

To determine the mechanisms by which of EVs and the SH3b2 domain activate signal transduction, experiments were performed in WT Mφ, and in murine Mφ with deleted surface and/or endosomal receptors (ΔTLR2, ΔTLR3, ΔTLR4, ΔTLR2/TLR4, ΔTLR9) or deleted signaling adaptor proteins (ΔTRIF, ΔTRIF/TRAM, ΔMyD88, ΔIRF3, ΔIRF7, **Supplementary Table S1**). Cells were incubated with 1.5 µg/mL of SH3b2 or EV10. PBS buffer or protein mock were used as controls. Using the expression of *Oas1b*, as reporter of the innate antiviral responses and *Il10*, it was found that the treatment of Mφ with EVs or SH3b2 showed a similar pattern of response (**Figure 4**). However, different pathways seem to modulate the expression of these genes. For *Oas1b*, it was found that MφΔTLR2/TLR4, Mφ ΔTLR4, Mφ and ΔTRIF/TRAM, resulted in significantly decreased responses to EVs as well as to SH3b2 (**Figure 5**, **6**). KO Mφ in TLR3 and TLR9 tested did not result in a significant effect on their responses to EVs or SH3b2 (**Figure 5**). Using the expression of *Il10*, it was found that the double knockout of MφΔTLR2/TLR4 significantly decreases the sensing of SH3b2 while the ΔMyD88 abolished the sensing of both EVs and SH3b2. In contrast, KO ΔIRF3, ΔIRF7 did not result in significant effect on the sensing of SH3b2 or EVs. These results are in agreement with our previous report where we showed that preventing the cellular uptake of EVs using endocytosis inhibitors decreased the cells expression of the antiviral genes in human pancreatic cells (13).

The impact of the impaired ability of the Mφ ΔTLR2/TLR4, Mφ ΔTRIF/TRAM, and ΔMyD88 to sense SH3b2 or EVs was evaluated in MNV-1 infection assays. We found that while the mutations tested resulted in cells that were less sensitive to MNV infection albeit not statistically significant (**Supplementary Figure S3**), Mφ ΔTRIF/TRAM, and Mφ ΔMyD88 responded like the infection control in the presence of EVs or SH3b2 (**Figure 5**). These results were interpreted to mean that SH3b2 and EVs signaling through TRIF/TRAM and MyD88 is required to achieve full protection to MNV infection and the expression of both OAS pathway and IL10 secretion is needed to achieve the highest protective effect.

### *L. johnsonii* N6.2 EVs and SH3b2 lower MNV titers *in vivo*

**Figure 7:**
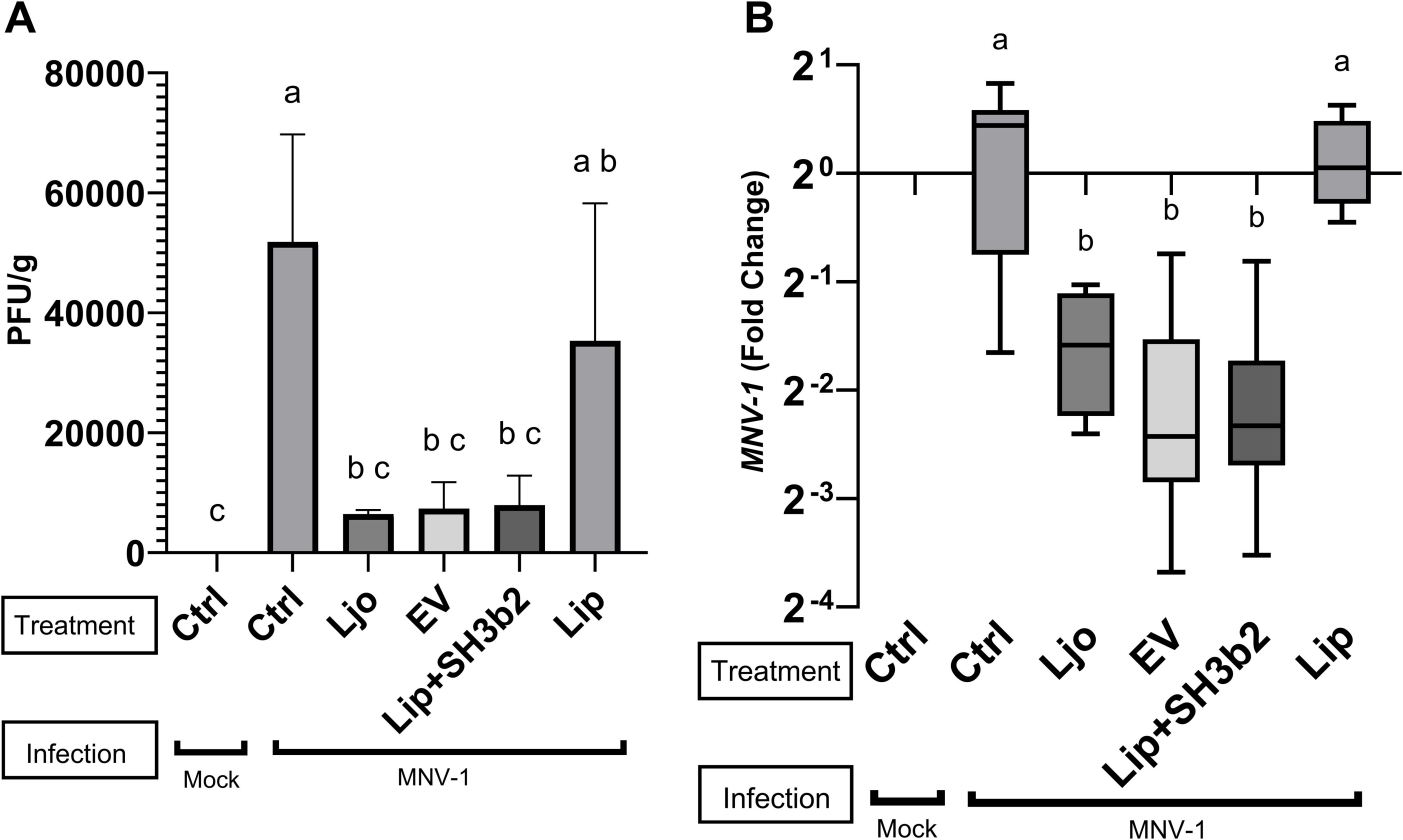
*In vivo* evaluation of the efficacy of EV, SH3b2 and *L. johnsonii* N6.2 on MNV-1 infection. Six-week-old C57BL/6 mice were orally administered *L. johnsonii* 10^8^ CFU, EV10, SH3b2, Liposomes or Liposomes loaded with SH3b2 daily for seven days (n=3/group). Mice were then infected with MNV-1 or mock virus for 24h. MNV was quantified by plaque assays **(A)** and by qRT-PCR **(B)**. Different letters on top of each bar indicates statistical significance of p ≤ 0.05 from ANOVA analysis and *post-hoc* Tukey test performed on three biological replicates.

C57BL/6 mice were orally fed with 20 µL of PBS containing *L. johnsonii* N6.2 at 1x10^8^ CFUs, EVs at 1x10^10^ particles, and 3 µg SH3b2. Additionally, 1x10^10^ liposomes made of *L. johnsonii* lipids as well as 1x10^10^ liposomes loaded with 3 µg SH3b2 were tested. PBS Buffer carrier was administered to the control group. After seven days, all groups were challenged with MNV-1 at 1x10^7^ viral particles per animal for 24 h. It was found that *L. johnsonii* N6.2, EVs and liposomes+SH3b2 significantly decreased MNV titers in the distal ileum while liposomes alone showed no significant effect on MNV-1 infection titer (**Figure 7**). In agreement with this observation, the mRNA MNV genome quantification from distal ileum tissue also showed a significant decrease in relative intracellular viral load. Taken together, these results show that *L. johnsonii* N6.2, EVs as well as SH3b2 can help in the control viral infections and to potentially decrease transmissibility by lowering the viral titers shed by the host.

## Discussion

Here, we report that *L. johnsonii* N6.2 EVs can mitigate replication of MNV-1 by stimulating the innate immune responses in host cells. Specifically, we found that the Sdp-SH3b2 domain can replicate the effects observed with EVs. While the antiviral effect of probiotics has been reported, our results significantly contribute to the identification of bacterial effector molecules that mediates these beneficial effects. Our study also found that *L. johnsonii* N6.2 EVs induce the expression of IL10 in murine RAW 246.7 Mφ, limiting the induction of pro-inflammatory IFNα and IFNβ compared to the MNV-1 control. We propose that the tolerogenic response resulting from IL10 stimulation is able to counteract the inflammatory response mediated by IFNs. These findings were complemented with the induction of *Ifi44l.* The immunomodulatory IFI44L plays a role in suppressing the cellular inflammatory response induced by cytokines (29). The Sh3b2 domain similarly induced IL10 expression, further indicating its role as a key effector in the overall immunomodulatory response.

The roles of live probiotic organisms in pathogen exclusion have been well-documented, particularly in the prevention of bacterial infections (19). However, this concept is not limited to the prevention of bacterial infections as microorganisms such as *Pediococcus pentosaceus* CAU170230-3 can reduce norovirus titers in fermented products like kimchi (30). There, it was proposed that viral titers are decreased through direct binding of the bacterium to virus particles. A similar mechanism was reported for *Enterobacter cloacae* and *Bacteroides thetaiotaomicron* (25). Other mechanisms reported include modification to binding cell receptors, such as changes in surface glycan motifs, needed for viral attachment induced by soluble factors produced by *Lacticaseibacillus casei* DN114 001 or *Bacteroides thetaiotaomicron* VPI-5482 (31). Additionally, Probiotic bacteria like *Bacillus subtilis*, *B. pumilus*, and *B. megaterium* can also enzymatically degrade virions structures necessary for viral adhesion and entry, like proteins and lipids, by secreting enzymes such as α-chymotrypsin and lipases (32).

Bacterial components, postbiotics, have been shown to induce the expression of cytokines in host cells, inhibiting viral infections. For example, exopolysaccharides of *Streptococcus thermophilus* ST538 induced the expression of *IL6* and *CCL2* in porcine intestinal epitheliocytes which could aid against a virus infections (33). Bacteriocins secreted by *Lactococcus lactis* subsp. *lactis* and *Enterococcus durans* can enhance the cellular antiviral response against herpes simplex virus 1 and poliovirus through undefined mechanisms (34). However, the use of purified bacterial vesicles for the treatment or prevention of viral infections *in vivo* is still an understudied field. Our study shows that *L. johnsonii* N6.2 EVs strongly stimulates innate immune responses, like an RNA viral infection, effectively reducing MNV-1 titers *in vitro* and *in vivo*. A comparable antiviral response has been observed with *Ligilactobacillus salivarius* HHuMin-U, by activating TBK1-IRF3 and NF-κB (35). However, the specific components involved in the activation on these pathways were not elucidated. The *in vitro* prevention of HIV infection of T-cells by EVs from *Lactobacillus* strains was proposed to be mediated by steric hindrance, by limiting viral entry in the cell (24). Contrary to these reports, our results show that *L. johnsonii* N6.2 EVs was effective against MNV-1 replication independent of the timing of the administration (pre-, co-, or post-infection) which may be explained by the rapid uptake of *L. johnsonii* N6.2 EVs previously reported (13).

One of the limitations of ongoing research to evaluate the role of probiotics and postbiotics in antiviral responses, is the lack of information on specific molecules that mediate these effects. Here, we identified Sdp and specifically its SH3b2 domain, enriched in *L. johnsonii* N6.2 EVs, as an effector peptide able to elicit a similar antiviral response as the native EVs. *L. johnsonii* Sdp consists of a N-terminal glycosyl hydrolase family 25 (GH25) domain, and six tandem repeats on the C-terminal side (9). These repeats display src-homology-3 (SH3) fold and are connected by short linker sequences. The GH25 domain is predicted to cleave glycosidic bonds in the peptidoglycan of bacterial cell walls. Domains of bacterial proteins, classified as SH3b, are homologues to the eukaryotic Scr-homology 3 (SH3) domain. SH3 domains in eukaryotic proteins are involved in signal transduction and binding of intra- or intermolecular proline-rich motifs (36). In general, SH3b domains have been associated with a variety of proteins, often with enzymatic activity (37). In *Staphylococcus aureus*, SH3b domains have shown to recognize peptidoglycans containing a pentaglycine crossbridge (38). Similar to eukaryotic members, the SH3 domain of the diphtheria toxin repressor, DtxR can bind to a flexible proline-rich peptide motifs in human proteins (39). We hypothesize that the SH3b domains of Sdp in *L. johnsonii* can moonlight as an effector molecule by binding to proline-rich motifs in eukaryotic proteins involved in signal transduction pathways. In support of this hypothesis, we have recently shown that *L. johnsonii* N6.2 EVs, promote Stat3 phosphorylation in Y705 followed by IL10 induction as well as AHR translocation (11). This proposed mechanism is further supported by recent reports from the pathogenicity factors SarA/SteE from *Salmonella* and YopM in *Yersinia enterocolitica* (40, 41). SarA mediates phosphorylation of Stat3, thereby inducing nuclear translocation and induction of IL10 (42). Similarly, YopM showed similar effect on Stat3 nuclear translocation to induce IL10 expression (41). However, whether physical interaction of these effector proteins, Sdp-SH3b2, YopM or SarA, with Stat3 results in activation is still unknown.

Herein we propose that EVs, through the Sdp-SH3b2 effector molecule initiate a variety of sensory pathways in Mφ leading to the stimulation of innate immune responses and decreased MNV-1 titers. The stimulation of IL10 expression has a pivotal role in the activation of the Jak/Stat pathway, leading to a fine-tunning of IFN-I responses (43). IFN-α/β are critical players to prevent the accumulation of non-structural proteins of MNV-1 blocking virion production (44). Here we observed that EVs can stimulate the expression of INF-beta in dose dependent manner which positively correlated with decreased MNV levels. However, the response obtained do not seem to be mediated through the canonical IRF3 or IRF7 transcription factors as the stimulation of KO Mφ on these transcription factors with EVs or Sdp-SH3b2 did not result in significant loss of function. We also observed that KO Mφ deficient in the sensory adaptors ΔTRIF/TRAM, and ΔMyD88 suppressed the induction of OAS pathway and IL10 expression, respectively, and that both are required for the protective role of EV. *In vivo* analysis of the *L. johnsonii* N6.2 EVs and the SH3b2 demonstrated a decrease in viral load compared to controls.

The results presented here provide further evidence that EVs from *L. johnsonii* N6.2 can mediate interkingdom signaling, by stimulating the innate immune response in the eukaryotic host to reduce viral replication. In the context of host:microbe interactions, EVs have been implicated in many mechanisms associated with their parent bacterial source including pathogenesis, host defense evasion and immune stimulation. Bacterial extracellular vesicles contain a unique composition of bioactive molecules that can elicit different effects in the host such as proteins and nucleic acids. The bacterial cell membrane biogenesis of Gram-positive derived EVs give them access to the whole cell protein, metabolite, and nucleic acid content. The EV cargo can then be trafficked from the parent bacteria to the target host cell eliciting a response quicker than a natural RNA viral infection that requires the expression of viral genome and proteins to reach critical levels before the cell can trigger an antiviral response. While the reduction in MNV-1 replicates observed when cells are treated by *L. johnsonii* N6.2 EVs *in vitro* is an important first step, further *in vivo* studies are required to validate our findings.

## Materials and methods

### Bacterial growth and EV isolation

*L. johnsonii* N6.2 was grown in exosome depleted de Man, Rogosa, Sharpe media (ED-MRS), previously described. Briefly, 500mL of the MRS media was ultracentrifuged, at 175,000 x *g* for 2 h at 4°C, to remove any media derived exosomes and extracellular vesicles that may be present in the yeast or beef extract, then filter-sterilized (0.2 µm). *L. johnsonii* N6.2 was inoculated into the ED-MRS at 1% (v/v) and incubated at 37°C under static condition to an OD_600_ = 1. After incubation, the 500 mL of *L. johnsonii* N6.2 cultures were centrifuged as 20,000 x *g* for 20 min, at 4°C and the supernatants were filter-sterilized (0.2 µm) to ensure removal of residual bacterial cells. The filtered supernatant was then ultracentrifuged at 175,000 x *g* for 2 h, at 4°C, to concentrate the EVs. The resulting EV pellets were washed twice with filtered PBS using the same ultracentrifugation parameters (9, 13). EVs suspended in PBS were subsequently quantified using a NanoSight 300 (Malvern instruments Ltd, Malvern, UK) at the University of Florida ICBR Flow Core Facility, RRID: SCR_019119.

### Murine norovirus production

Murine norovirus MNV-1 was produced as previously described (25, 45, 46). Briefly, the pSPMNV-1. CW3 plasmid (5 μg) was transfected into HEK 293T cells to express the recombinant murine norovirus-1 genome. The supernatant from transfection (MOI=0.05) was used to infect RAW264.7 cells. At 36-48 hours post-infection (hpi), the cells were checked for ~90% cytopathological effect. Supernatant containing viruses was harvested ultracentrifuged through a 25% sucrose cushion to obtain purified viral pellets. Viral pellets were resuspended in PBS and titrated using TCID_50_ assay. All virus stocks were aliquoted and stored at -80°C upon receipt and were thawed on ice for 1 h prior to use. Mock inoculum was generated by transfecting HEK 293T cells with the pSP.CW3 plasmid not containing the MNV-1 genome and following the same viral isolation procedure stated above.

### Murine cell lines propagation

All cell lines were grown in complete growth media containing Dulbecco’s Modified Eagle Medium (DMEM) (4.5 g/L glucose, 4mM L-glutamine, and 1 mM sodium pyruvate) supplemented with 10% heat inactivated FBS (Sigma-Aldrich, Saint Louis, MO, USA), 1% penicillin and streptomycin solution containing 10,000 units of penicillin and 10 mg of streptomycin/mL (Sigma-Aldrich, Saint Louis, MO, USA). Initial experiments and viral challenge were measured in the murine Mφ cell line RAW 264.7. To further elucidate the mechanisms by which EVs initiate the host sensory response, knockout murine Mφs cell lines were used. The following reagents have been obtained from BEI Resources, NIAD, NIH: Mφ cell line derived from wild type mice NR-9456 (WT), and the Mφ cell lines derived from following knockout Mice: ΔTLR2 (NR-9457), ΔTLR3 (NR-19974), ΔTLR4 (NR-9458), ΔTLR2/TLR 4 (NR-19975), ΔTLR7 (NR-15634), and ΔTLR 9 (NR-9569). The following adaptor proteins will also be evaluated: Mφ cell line derived from: ΔTRIF/TRAM (NR-9568), ΔMyD88 (NR-15633), ΔMAL (NR-9459); as well as the ΔIRF3 (NR-15635), and ΔIRF7 (NR-15636) (BEI). RAW-DUAL KO-TLR4 reporter cell line was obtained from (InvivoGen, San Diego, CA) See **Supplementary Table S1**.

### Inoculation of Mφ cell lines with MNV-1 and *L. johnsonii* N6.2 EVs

Mφ cell lines were plated 5x10^5^ cells/mL in a 12 well plate and grown for 48 h to reach confluence (~1x10^6^ cells). For the treatments, *L. johnsonii* N6.2 EVs were added at final increasing concentrations as follows: 1 x 10^8^ (EV8), 1 x 10^9^ (EV9), 1 x 10^10^ (EV10) EVs in DMEM media. The Mφ cells were either pre-treated, EVs were added 5 h prior to virus inoculation, co-inoculated with MNV-1, or the EVs were added 1 h after the virus inoculation. The MNV-1 inoculum was prepared as 500 μL solution of MNV-1 (MOI=5) in DMEM media and incubated at 37°C for 1 h with gentle rotation prior to infection. Plated cells were then inoculated with 500 μL of the MNV-1 solution into each well then rocked gently to mix and incubated at 37°C with 5% CO_2_ for 1 h. The inoculum was removed from each well, and wells were washed with PBS twice. Complete media was added to the wells, and the cells were grown for 6 or 18 hpi at 37°C with 5% CO_2_. The supernatant was then collected for lactate dehydrogenase (LDH) analysis, MNV-1 quantification, and cells were lysed for RNA isolation and qRT-PCR analysis.

### mRNA extraction and qRT-PCR

RNA was extracted from eukaryotic cell lines using the Qiagen RNeasy Miniprep following manufacture’s specifications (QIAGEN, Germantown, MD). The RNA extracted were treated with DNase using the DNase Turbo kit (Thermo Scientific, Waltham, MA). qRT-PCR was performed using a QuantStudio 6 Flex (Thermo Scientific, Waltham, MA) as previously described (13). The expression of *GAPDH* was used as the endogenous control. The sequences of the primers used are listed in **Supplementary Table S2**. MNV-1 genome was quantified by amplifying the MNV-1 cDNA genome using specific primers for the ORF1 MNV-1 genome. MNV-1 levels were expressed as either relative expression compared to a control condition or absolute MNV-1 genome counts by creating a standard curve with the pSPMNV-1 plasmid.

### Lactate dehydrogenase assay

Viral induced cytotoxicity was evaluated in Mφ cell lines by following the leakage of the cytoplasmic enzyme Lactate Dehydrogenase (LDH) to the supernatant. Supernatant LDH activity was measured using the CyQUANT LDH Cytotoxicity assay kit (Thermo Scientific, Waltham, MA) following manufacturer’s specification. The percent difference (Δ%) was calculated by normalizing to the control group not treated with either the MNV or *L. johnsonii* N6.2 EVs.

### Mice infection

C57BL/6 mice were housed in the University of Florida animal facilities. The animal protocols were approved by the Institutional Animal Care and Use Committees at the University of Florida. The mice were orally fed daily for seven days. The treatments (N=3) were as follow: PBS for the mock and MNV only groups, 1x10^8^ colony forming units (CFU) of *L. johnsonii* N6.2, 20 µL PBS containing 1x10^10^ EVs, 20 µL of PBS containing 1x10^10^ liposomes, 20 µL PBS containing 3 µg of SH3b2, and 20 µL PBS containing 1x10^10^ liposomes loaded with 3 µg of SH3b2. On the seventh day, all groups of mice (except the mock group) were orally inoculated with 1x10^7^ MNV-1 or mock inoculum. 24 hours post infection (hpi) mice were euthanized using a CO_2_ fill rate of 30-70% of the chamber volume per minute until breathing stop followed by decapitation. Viral titers were analyzed in distal ileum tissues by qRT-PCR as described earlier as well as by plaque assay (45). The experiment was repeated twice.

### Gene overexpression and protein purification

The following proteins enriched in EVs were cloned: Sdp (locus tag T285_RS00825, domains SH3b2, SH3b6, SH3b1-SH3b2, and Lys), Muc (locus tag T285_RS08930, domains Muc1, Muc3, Muc4, Muc5), LexA, PepD, PepC, P8875 and Eno3. Standard methods were used for *L. johnsonii* N6.2 genomic DNA isolation (QIAGEN DNeasy Blood and Tissue Kit, Germantown, Maryland, USA), restriction enzyme digestion, agarose gel electrophoresis, ligation and transformation (47, 48). The primers used are listed in **Supplementary Table S2**. PCR fragments were obtained and cloned into p15TVL (GenBank accession EF456736) vector. Upon transformation into *Escherichia coli* DH5α, recombinant plasmids were confirmed by sequencing with T7 universal primers. His-tagged fusion genes were transformed into *E. coli* BL21 (DE3). For protein purification, cells were grown in Luria Broth at 37°C to an optical density of 0.8. Genes were overexpressed with 0.5 mM isopropyl-tiol-β-D-galactopyranoside (IPTG) and incubated at 17°C for 16 h. Cells were harvested by centrifugation at 7,800 x *g* for 20 min. Next, the cell pellet was resuspended in binding buffer (500 mM NaCl, 5% glycerol, 50 mM Tris, 5 mM imidazole, pH 8.0) and lysed using a French press. The lysates were then centrifuged at 35,000 x *g* for 45 min and the supernatant was applied to a Ni^2+^ affinity column. The column was washed with 200 mL of wash buffer (binding buffer with 25 mM imidazole) and the proteins were eluted with elution buffer (binding buffer with 250 mM imidazole). The purified proteins were dialyzed against 10 mM Tris (pH=8), 2.5% glycerol, 500 mM NaCl and 0.5 mM TCEP, and stored at -80°C (47, 48). Protein concentrations were measured by Bradford assay (Bio-Rad). Bovine serum albumin (BSA; Gold BioTechnology, St. Louis, MO, USA) was used as the standard. Mock was prepared following a protein purification protocol using *E. coli* BL21 (DE3) not containing an expression vector.

### Secreted embryonic alkaline phosphatase and Lucia luciferase assays

RAW-DUAL KO-TLR4 reporter cell line (InvivoGen, San Diego, CA) was treated in triplicates with EVs at 1:10,000 ratio or LPS with 100 ng/mL as the control. After 24 h, cell culture supernatants were collected and analyzed. SEAP expression is dependent on the activation of the endogenous MIP-2 promoter. MIP-2 expression is NF-κB dependent and the SEAP gene replace the MIP-2 open reading frame (ORF). The expression of the Lucia luciferase gene is under the control of the ISG54 promoter in conjunction with five IFN-stimulated response elements (ISRE). Both the SEAP and Lucia luciferase reporter proteins are secreted and measurable from the cell culture supernatant. SEAP and Lucia luciferase activity were measured by a microplate photometer (BioTek, USA) using QUANTI-Blue solution (InvivoGen, USA) and QUANTI-Luc 4 Lucia/Gaussia solution (InvivoGen, USA) to detect SEAP and Lucia luciferase activity, respectively, following manufacture’s protocol.

### Bacterial lipid extraction and liposome formation

*L. johnsonii* N6.2 cultures were grown and harvested as described above. Cells were washed twice with 1% (w/v) NaCl then frozen at -80°C overnight and freeze-dried (Labconco, Kansas City, MO, USA) for 24h. Total lipids from *L. johnsonii* N6.2 were extracted using a modified Bligh and Dyer method as previously reported (13, 49). The dried total lipid extract was then resuspended in 1 mL of 1x PBS. The PBS lipid suspension is then extruded through a 0.1µm polycarbonate membrane filter in an Avanti mini extruder following manufacturer’s protocol (Avanti Polar Lipids, AL, USA). For liposomes containing SH3b2, the quantified extruded liposomes were extruded again with the desired protein amount and quantified using Nanosight as described above.

### Statistical analysis

GraphPad Prism 9.491 software (GraphPad Software, La Jolla, CA, United States) and Origin 9.7.0.188 (OriginLab Corporation, Northampton, MA, USA) were used for data analysis and visualization. Statistical tests were performed using one-way analysis of variance (ANOVA) to evaluate the effects of treatments, followed by a Tukey *post-hoc* test. Results were summarized as means ± standard deviation, and significance of model terms and treatment comparisons were considered significant at a level of α=0.05.

## Data Availability

The datasets generated for this study are available upon request to the corresponding author.
