# Supplementary material for: The Sdp-SH3b2 domain contained in Lactobacillus johnsonii N6.2-derived extracellular vesicles inhibit murine norovirus replication
Source: Front Immunol. 2024 Dec 5;15:1490755. doi: 10.3389/fimmu.2024.1490755 (PMC11659762; doi:10.3389/fimmu.2024.1490755)
Supplement: Supplementary file 1 [file DataSheet1.pdf]

Supplementary Table 1. Bacterial and Macrophage cell lines utilized.

**Bacterial Strains**	**Genotype**	**Reference or Source**
Lactobacillus johnsonii N6.2	Wild type	Laboratory stock[46]
Escherichia coli DH5α	*F*$^{-}$ *Φ80lacZΔM15 Δ(lacZYA-argF)U169 recA1 endA1 hsdR17 (rK*$^{-}$ *mK*$^{+}$*) phoA supE44 λ*$^{-}$ *thi-1 gyrA96 relA1*	Invitrogen
E. coli BL21 (DE3)	*F*$^{-}$ *ompT hsdS(rB*$^{-}$ *mB*$^{-}$*) gal dcm (DE3)* pRARE	Novagen
E. coli EESH3b1-SH3b2	BL21 (DE3) carrying p15TV- Sdp-SH3b1-SH3b2 (Amp^r)	*
E. coli EESH3b2	BL21 (DE3) carrying p15TV- Sdp-SH3b2 (Amp^r)	[9]
E. coli EESH3b6	BL21 (DE3) carrying p15TV- Sdp-SH3b6 (Amp^r)	[9]
E. coli EELys	BL21 (DE3) carrying p15TV- Sdp-Lys (Amp^r)	*
E. coli EEMuc1	BL21 (DE3) carrying p15TV- Muc1 (Amp^r)	*
E. coli EEMuc3	BL21 (DE3) carrying p15TV- Muc3 (Amp^r)	*
E. coli EEMuc4	BL21 (DE3) carrying p15TV- Muc4 (Amp^r)	*
E. coli EEMuc5	BL21 (DE3) carrying p15TV- Muc5 (Amp^r)	*
E. coli EEEno3	BL21 (DE3) carrying p15TV- Eno3 (Amp^r)	[9]
E. coli EELexA	BL21 (DE3) carrying p15TV- LexA (Amp^r)	[9]
E. coli EEpepD	BL21 (DE3) carrying p15TV- PepD (Amp^r)	*
E. coli EEPepC	BL21 (DE3) carrying p15TV- PepC (Amp^r)	*
E. coli EEP8875	BL21 (DE3) carrying p15TV- P8875 (Amp^r)	*
**Macrophage (Mφ) cell lines**		
Wild type Mφ (WT) RAW 264.7	*Mus musculus* Mφ cell line	ATCC
RAW-Dual™ ΔTLR4	TLR4 knockout murine Mφ. IRF and MIP-2 (NF-κB) reporter (IRF54Lucia/KI; MIP-2:SEAP).	InvivoGen
WT Mφ NR-9456	Murine Mφ cell line derived using primary bone marrow cells from wild type mice and immortalized by infection with the ecotropic transforming replication-deficient retrovirus J2	BEI Resources
ΔTLR2 Mφ NR-9457	Derived from TLR2 (toll-like receptor 2) knockout (KO) mice	BEI Resources
ΔTLR3 Mφ NR-19974	Derived from TLR3 (toll-like receptor 3) KO mice	BEI Resources
ΔTLR4 Mφ NR-9458	Derived from TLR4 (toll-like receptor 4) KO mice	BEI Resources
ΔTLR2/TLR4 Mφ NR-19975	Derived from TLR2 (toll-like receptor 2)/TLR4 (toll-like receptor 4) double KO mice	BEI Resources

ΔTLR9 Mφ NR-9569	Derived from TLR9 (toll-like receptor 9) KO mice	BEI Resources
ΔTRIF/TRAM Mφ NR-9568	Derived from TRIF (toll-interleukin-1 receptor domain-containing adaptor-inducing interferon-β)/TRAM (TRIF-related adaptor molecule) double KO mice	BEI Resources
ΔMyD88 Mφ NR-15633	Derived from MyD88 (myeloid differentiation primary response protein 88) KO mice	BEI Resources
ΔIRF3 Mφ NR-15635	Derived from IRF3 (interferon regulatory factor 3) KO mice	BEI Resources
ΔIRF7 Mφ NR-15636	Derived from IRF7 (interferon regulatory factor 7) KO mice	BEI Resources

*Original to this work

Supplementary Table 2 Primers utilized for qRT-PCR.

Primer name	Primer sequence (5'-3')	Source
murine *Gapdh* Forward	AGTATGACTCCACTCACGGCAAAT	*
murine *Gapdh* Reverse	GTCTCGCTCCTGGAAGATGGT	*
murine *Oas1b* Forward	TGGACCTAGGATGGAGCAGG	*
murine *Oas1b* Reverse	GCACACAGGGCATCTATGACT	*
murine *Oas2* Forward	GGCTGTCCTCCCCTTGTTTC	*
murine *Oas2* Reverse	CACTCCCACCATGTCACTTGT	*
murine *Oasl1* Forward	CAGCGAAACTTCGTGAAGCA	*
murine *Oasl1* Reverse	CAGGATGATAGGCCTGCTGGT	*
murine *Mx1* Forward	CAGAAAGCCAAGCACTCTGTC	*
murine *Mx1* Reverse	ATGTTTTCCAGGAGGATTCCAGT	*
murine *Mx2* Forward	AGCTGCAGAAGTACGGTATGG	*
murine *Mx2* Reverse	GACATTCTCCCTCTGCCACAT	*
murine *Ifi44l* Forward	AAAGAAGATGGTGACCGCCG	*
murine *Ifi44l* Reverse	AAGCTCGACTTTCCCTGAGC	*
murine *Ifna1* Forward	CTCAAGCCATCCCTGTCCTG	*
murine *Ifna1* Reverse	TCTTCCTGGGTCAGGGGAAA	*
murine *Ifna4* Forward	AAAGCCTGTGTGATGCAGGA	*
murine *Ifna4* Reverse	TGGTTGAGGAAGAGAGGGCT	*
murine *Ifnb* Forward	AACTCCACCAGCAGACAGTG	*
murine *Ifnb* Reverse	GGTACCTTTGCACCCTCCAG	*
murine *Ifnl2* Forward	GAAGGTCTGGGAGAACATGACT	*
murine *Ifnl2* Reverse	GGAGTGAATGTGGCTCAGTGT	*
murine *Ifnl3* Forward	TGCAGTTCCCACCTCATCTC	*
murine *Ifnl3* Reverse	GTCTGCAGCTGGGAGTGAAT	*
murine *Il10* Forward	TGGGTTGCCAAGCCTTATCG	*
murine *Il10* Reverse	CAGCTTCTCACCCAGGGAAT	*
MNV-1 Forward	GTGCGCAACACAGAGAAACG	[25]
MNV-1 Reverse	CGGGCTGAGCTTCCTGC	[25]

*Original to this work

Supplementary Table 3. Primers used amplification of genes from *L. johnsonii* N6.2

Locus tag	Primer name	`	Source
T285_RS00825	SH3b2 Fw	TTGTATTTCCAGGGCCCAAGTACAAACACTAACGTAAATA	[9]
T285_RS00825	SH3b2 Rv	CAAGCTTCGTCATCACTTTTCTGCTGGTTGACTT	[9]
T285_RS00825	SH3b6 Fw	TTGTATTTCCAGGGCGCTAATAAGCCTGTTCGACAAA	[9]
T285_RS00825	SH3b6 Rv	CAAGCTTCGTCATCATTATCTGTAAGTTCCCCATGCTT	[9]
T285_RS00825	SH3b1-SH3b2 Fw	TTGTATTTCCAGGGCCCAAACACAACCACTAATGTAAA	*
T285_RS00825	SH3b1-SH3b2 Rv	CAAGCTTCGTCATCACTTTTCTGCTGGTTGACTT	*
T285_RS00825	Lys Fw	TTGTATTTCCAGGGCACTGATACTGTACCTGCAAAGTC	*
T285_RS00825	Lys Rv	CAAGCTTCGTCATCAAGCAGCGTTATTTGAGGTGTT	*
T285_RS08930	Muc1 Fw	TTGTATTTCCAGGGCGGACTTAGTGCTATTTTGCCTGA	*
T285_RS08930	Muc1 Rv	CAAGCTTCGTCATCAAGCACTTACAGCTGCACCATT	*
T285_RS08930	Muc3 Fw	TTGTATTTCCAGGGCGTAGCGCAAGGCTCAAGAAC	*
T285_RS08930	Muc3 Rv	CAAGCTTCGTCATCATGTTGGATAAGTTGGGGTTGG	*
T285_RS08930	Muc4 Fw	TTGTATTTCCAGGGCACTCAGCGTGCTAGTCACCA	*
T285_RS08930	Muc4 Rv	CAAGCTTCGTCATCATGGACCTACTGGAATTTGGTT	*
T285_RS08930	Muc5 Fw	TTGTATTTCCAGGGCAAGATTGGTAAGATAGTTCCAGTTGA	*
T285_RS08930	Muc5 Rv	CAAGCTTCGTCATCA TTGCTTAACTGGTTGTTCAGGT	*
T285_RS03880	Eno3 Fw	TTGTATTTCCAGGGCATGCTCAAATCAGTTATTGAG	[9]
T285_RS03880	Eno3 Rv	CAAGCTTCGTCATCATTAATCTAAGTCAACGTTGTC	[9]
T285_RS03645	LexA Fw	TTGTATTTCCAGGGCATGACTGAACCACATGCAAA	[9]
T285_RS03645	LexA Rv	CAAGCTTCGTCATCATTAATCAATATTATTACGATATAGACC	[9]
T285_RS01325	PepD Fw	TTGTATTTCCAGGGCATGAAACCAACCGAATGTAC	*
T285_RS01325	PepD Rv	CAAGCTTCGTCATCATTAATCAAGTAAGTCATATTTTAAGG	*
T285_RS01810	PepC Fw	TTGTATTTCCAGGGCATGAGCAAAGAAATCACAAATGATC	*
T285_RS01810	PepC Rv	CAAGCTTCGTCATCACTAATAATTATTAGCTAAAGCACCC	*
T285_RS08570	P8875 Fw	TTGTATTTCCAGGGCATGAAAATTTTAGTAACAGGCTTTG	*
T285_RS08570	P8875 Rv	CAAGCTTCGTCATCATTAGTGATTTGCTCCCTCA	*

*Original to this work

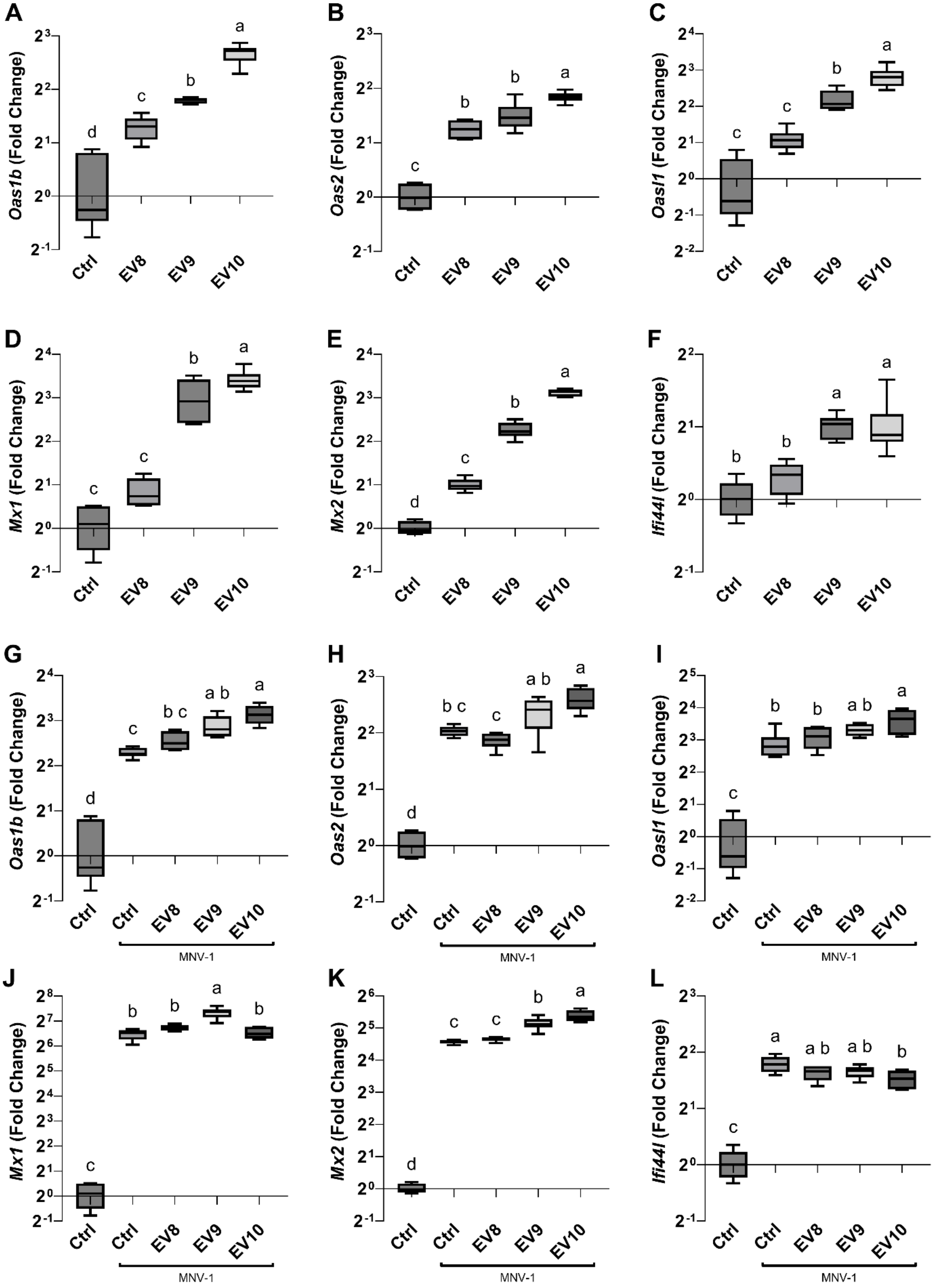
A
Oas1b (Fold Change)
Ctrl
EV8
EV9
EV10
B
Oas2 (Fold Change)
C
Oasl1 (Fold Change)
D
Mx1 (Fold Change)
E
Mx2 (Fold Change)
F
Ifi44l (Fold Change)
G
H
I
J
K
L
MNV-1

Supplementary Figure 1. Dose-dependent activation of antiviral genes in RAW 264.7 cells by *L. johnsonii* N6.2 EVs after 6 h of incubation (A-F) or after MNV-1 infection (18 hpi) (G-L). qRT-PCR analysis of the murine genes (A, G) *Oas1b*, (B, H) *Oas2*, (C, I) *Oasl1*, (D, J) *Mx1,* (E, K) *Mx2* and (F, L) *Ifi44l.* Different letters on top of each bar indicates statistical significance of $p \leq 0.05$ from ANOVA analysis and post-hoc Tukey test performed on three biological replicates (with two qRT-PCR technical replicates each).

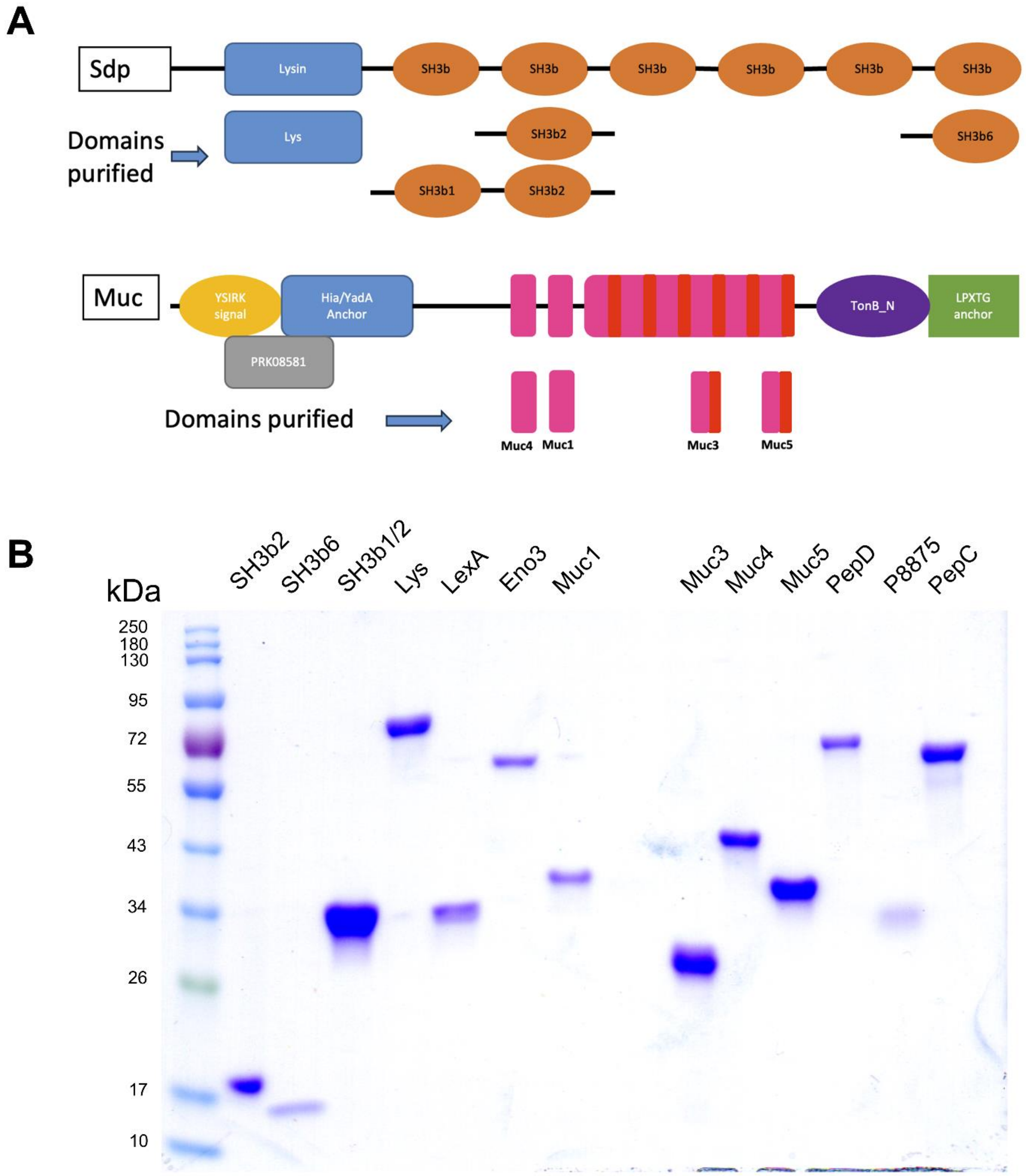

Supplementary Figure 2. (A) Schematic representation of the location of the repetitive domains selected in Sdp and Muc. (B) SDS PAGE of the purified proteins.

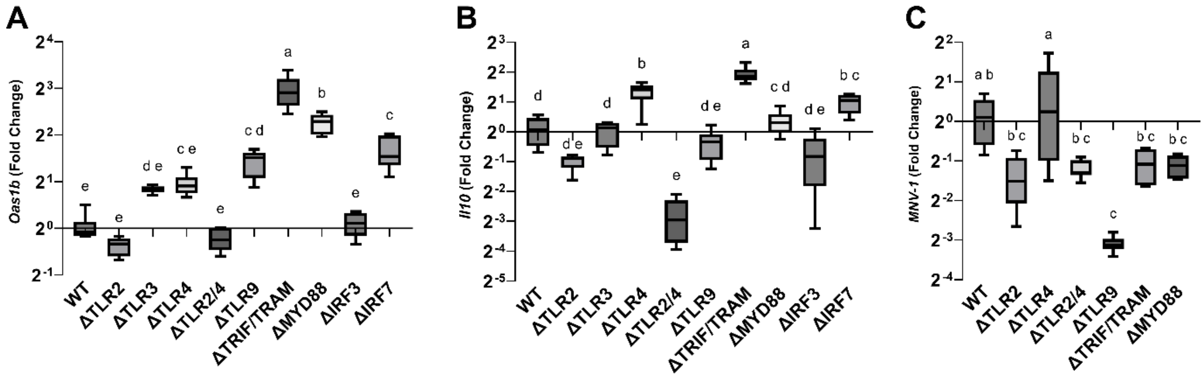

Supplementary figure 3. Differential expression levels of *Oas1b* (A) and *Il10* (B) in all the Mφ cell lines tested. The expression of the mRNA levels of *Oas1b* and *Il10* genes was normalized to the Wild type (WT) Mφ cell line. C) Effect of the deletions of cell receptors and pathway adaptor proteins on MNV-1 infection capabilities. The MNV-1 genome titer was quantified 18 hpi by qRT-PCR. Values were normalized t the WT Mφ cell line. Different letters on top of each bar indicates statistical significance of $p \leq 0.05$ from ANOVA analysis and post-hoc Tukey test performed on three biological replicates (with two qRT-PCR technical replicates each).

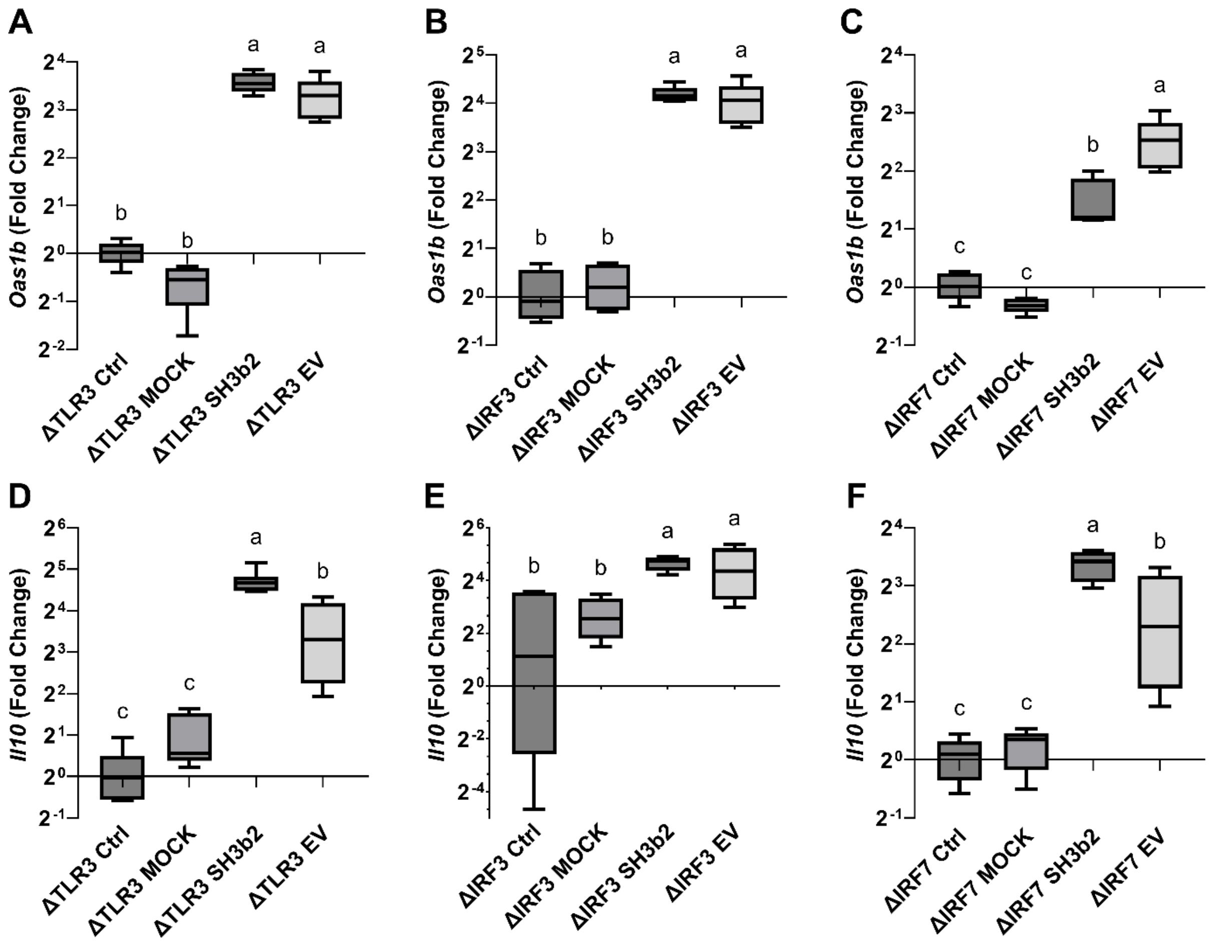

Supplementary Figure 4. SH3b2 and EVs stimulation is not affected in Mφs derived from knockout mice the ΔTLR3, ΔIRF3 or ΔIRF7 Mφ cell lines. Purified SH3b2 (1.5 μg/ml), EV10, and purification MOCK and buffer controls were added to immortalized murine Mφ cells. The stimulation of the expression of the mRNA levels of *Oas1b* and *Il10* genes was evaluated after 6 h in ΔTLR3 (A, D), ΔIRF3 (B, E) or ΔIRF7 (C, D), respectively. Different letters on top of each bar indicates statistical significance of $p \leq 0.05$ from ANOVA analysis and post-hoc Tukey test performed on three biological replicates (with two qRT-PCR technical replicates each).
